# Potential eating disorder exhibited with daytime functional vulnerabilities associated with sleep problems in Japanese adolescents: A cross‐sectional study

**DOI:** 10.1002/brb3.2605

**Published:** 2022-05-09

**Authors:** Takaharu Hirai, Yuta Mitobe, Hiromi Hirai, Momoka Takeda, Mikiko Hayashi

**Affiliations:** ^1^ Department of Psychiatric and Mental Health Nursing University of Fukui Fukui Japan; ^2^ Graduate School of Health and Welfare Science International University of Health and Welfare Tokyo Japan; ^3^ Faculty of Nursing and Social Welfare Science Fukui Prefectural University Fukui Japan; ^4^ Fukui Prefectural Shimizu School for Special Needs Education Fukui Japan; ^5^ Fukui Prefectural Echizen City Shirayama Elementary School Fukui Japan

**Keywords:** adolescents, Athens Insomnia Scale, Eating Attitudes Test‐26, mental health, physical functioning, potential eating disorders, sleep problems, well‐being

## Abstract

**Objectives:**

Eating disorders (ED) are serious psychiatric disorders that affect individuals, especially adolescents. It has been suggested that nonclinical ED‐like characteristics are related to sleep problems. We conducted a survey of Japanese adolescents to investigate this claim.

**Method:**

In this cross‐sectional study, 398 adolescents aged 12–18 years responded to a self‐administered questionnaire survey. We used the Eating Attitudes Test‐26 (EAT‐26) and the Athens Insomnia Scale (AIS) to measure potential ED and sleep problems, respectively.

**Results:**

Adolescents with potential ED had significantly higher daytime functional vulnerability potentially associated with sleep problems than those without ED. In particular, problems with a sense of well‐being and physical and mental functioning during the day were significant. In contrast, no significant associations were found between potential ED and sleep initiation, awakenings during the night, early morning awakening, total sleep duration, or overall quality of sleep. Finally, nocturnal and daytime sleep scores were significantly associated with dieting, bulimia, and oral control EAT‐26 subscores.

**Discussion:**

Participants with possible ED experienced problems related to well‐being and mental and physical functioning, which are indicators of daytime functional vulnerability potentially associated with sleep problems. Further, adolescents with more severe ED characteristics are more likely to have a higher degree of daytime psychological vulnerability potentially attributable to sleep problems. The study suggested that professionals treating adolescent mental health issues need an approach that comprehensively integrates both sleep problems and potential ED.

## Graphical abstract

Adolescents with potential eating disorders (ED) had significantly higher daytime functional vulnerability potentially associated with sleep problems than those without ED. In particular, problems with a sense of well‐being and physical and mental functioning during the day were significant.

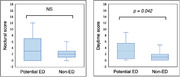

## 1 INTRODUCTION

Eating disorders (ED), characterized by a pathological distortion of eating habits and behaviors (American Psychiatric Association, 2013), are serious psychiatric disorders affecting many adolescents (Abebe et al., 2012; Stice et al., 2009), and ED risk often increases in mid‐ to late adolescence (Fairweather‐Schmidt & Wade, 2015). Symptoms at this age may not necessarily be severe enough to warrant a diagnosis of ED but include ED‐like characteristics such as body dissatisfaction and disordered eating behaviors (Neumark‐Sztainer et al., 2011). ED‐like characteristics are linked to developing ED later on (Littleton & Ollendick, 2003; McFarlane et al., 2001).

Previous studies have identified numerous biological, psychological, and sociocultural factors contributing to potential ED (Bulik et al., 2007, 2016; McCabe & Ricciardelli, 2003), including age, sex, and body mass index (BMI) (Holt & Ricciardelli, 2002; Rosenvinge & Pettersen, 2015). Sleep problems have also been reported to be associated with ED (Bos et al., 2013). Recent research using the Structural Sleep Disorders Questionnaire and all‐night polysomnography found that patients with anorexia and bulimia exhibit insomnia, daytime hypersomnolence, and parasomnias, with longer sleep latency, reduced sleep efficiency, and increased arousal index (Asaad et al., 2018). Nonclinical adolescents are particularly vulnerable to sleep problems and ED‐like behaviors (Carskadon, 2011; Neumark‐Sztainer et al., 2011). In a study of 13‐ to 18‐year‐olds in the United States (*n *= 6483), about a third of participants reported insomnia symptoms, indicating that those with insomnia had 3.4 times higher odds of having a mental disorder, including ED (Blank et al., 2015). A study of Portuguese students aged 17–25 years (*n *= 870) reported that disordered eating behavior predicted sleep problems at 1 (*n *= 592) and 2 (*n *= 305) years (Bos et al., 2013). Regarding the relationship between ED or disordered eating behavior and sleep problems, malnutrition was suggested to cause dysregulation of the neuropeptide orexin receptors in a clinical group of persons with anorexia nervosa, leading to secondary sleep problems (Allison et al., 2016). Another hypothesis is that insufficient sleep may increase cortisol levels, enhancing hunger and feeding behaviors and increasing susceptibility to ED (Trace et al., 2012). However, the underlying mechanism is not clear. In any case, sleep problems and eating disorder problems in adolescents are interrelated. In particular, these problems are likely to be more pronounced in the nonclinical adolescent (Gradisar et al., 2011; Neumark‐Sztainer et al., 2011).

Studies exploring the association between potential ED and sleep disorders have primarily been conducted in Europe and the United States. We were unable to find any studies on Asian adolescents, which would be representative of Japan. Customs surrounding eating habits, living arrangements, religion, and family differ between Japan and the West. There is also considerable variation in views about body image and the phenotype of ED. A previous study suggested that Japanese females have significantly higher levels of bulimic tendencies than Polish females (average age: 22.4 years old), and that the intensity of body dissatisfaction among the Japanese women (average age: 20.7 years old), unlike their Polish counterparts, was explained mainly by emotional dysregulation and the direct pressure of sociocultural standards of body image promoted by mass media (Izydorczyk et al., 2020). Furthermore, a study of Japanese (13‐ to 14‐year‐olds) and Finnish females (13‐ to 15‐year‐olds) suggested different associations between peer and prosocial behavior, body image, and eating distress (Maezono et al., 2019). Considering that cultural background may also influence ED and potential ED (Pike et al., 2014), it is meaningful to examine the association between potential ED and sleep problems in Japan, a non‐Western country. Increasing the knowledge related to potential ED may assist in screening adolescents for potential ED. Considering the existence of other specified feeding or eating disorders (OSFED) and the recent diversification of ED diagnoses, there is a risk of overlooking adolescents with potential ED when using existing screening methods. For example, the Eating Attitudes Test‐26 (EAT‐26), which is widely used in ED research in community populations and has been translated into Japanese (Mukai et al., 1994), has a high false‐negative rate in detecting bulimia nervosa (Nakai, 2003). The Eating Disorder Inventory‐2, used to screen for ED, has not been validated for undifferentiated ED assessments at least since the fourth edition of *Diagnostic and Statistical Manual of Mental Disorders* (Mintz & O'Halloran, 2000). Therefore, it is worthwhile to examine potential ED and sleep problems in the interest of expanding our screening capabilities.

This study examined the association between sleep problems and ED symptoms in Japanese adolescents, with the intention of increasing knowledge of potential ED and providing insight for future ED‐prevention strategies.

## 2 METHOD

### 2.1 Participants

We conducted a cross‐sectional study by listing all schools in the city of Niigata and using random number tables to randomly select four middle schools and four high schools. Data were collected from October 2017 to January 2018. All students in the selected schools were invited to participate in this study, and those who participated completed the questionnaire during the break between classes. We provided a clear written explanation of the study's objective and methods and obtained participants’ consent. Upon completion of the survey, participants enclosed their questionnaires in collection envelopes and deposited them in a collection box. The statistical analyses only included students aged ≤18 years. The participants comprised 398 students who answered all the items in the anonymous self‐administered questionnaire. According to a power calculation using G‐power 3.1 (Faul et al., 2007), a sample size of 398 participants (group 1 = 381, group 2 = 17) and a *p*‐value of 0.05 assure a power of 0.71, considering a mean effect size of *d* = 0.64 for the between‐group comparison of the primary outcome of sleep problems.

### 2.2 Main outcome variables

#### 2.2.1 Clinical characteristics

The questionnaire included the clinical characteristics of sex, age, height, and weight. For height and weight, participants were asked to self‐report the values at the time of completing the questionnaire. The provided values for height and weight were used to calculate BMI. BMI percentile is based on the 50th percentile by BMI sex and age.

#### 2.2.2 Eating Attitudes Test‐26

The Japanese version of the EAT‐26 was used as a preliminary screening tool to confirm potential ED in participants (Mukai et al., 1994). The EAT‐26 consists of 26 items divided into three subscales. The first subscale, "Dieting score," comprises 13 items related to distorted body image; the second subscale, "Bulimia score," comprises six items related to body image and tendency toward bulimic behavior; and the third subscale, "Oral Control score," comprises seven items on self‐control and high‐risk behaviors associated with anorexia nervosa. Each item is evaluated on a six‐point scale as "always," "very often," "often," "sometimes," "rarely," and "never." The first three responses are scored as 3, 2, and 1 point(s), respectively, and the last three are all assigned 0 points. The scores for all 26 items were summed to calculate the total EAT‐26 score, and the cutoff value was set at 20 points, in accordance with the original (Garner & Garfinkel, 1979; Garner et al., 1983) and Japanese versions of the EAT‐26 (Mukai et al., 1994). A higher score indicates more severe ED trends. In this study, participants with a total score of ≤19 points were classified as non‐ED, while those with scores of ≥20 points were classified as potential ED. Previous research using the EAT‐26 Japanese version found Cronbach's *α* coefficients of .79 for high school boys and girls (Mukai et al., 1994). In the present study, the *α* value was .83.

#### 2.2.3 Athens Insomnia Scale

Sleep problems were evaluated using the Japanese version of the Athens Insomnia Scale (AIS) (Okajima et al., 2013). It comprises eight questions intended to measure sleep initiation, awakenings during the night, early morning awakening, sufficiency of total sleep duration, overall quality of sleep, sense of well‐being during the day, functioning (physical and mental) during the day, and sleepiness during the day. While the first five items assess nocturnal sleep problems, the last three pertain to daytime sleep‐related problems. Each item is scored on a four‐point scale from "extremely problematic" to "no problem." Responses of "extremely problematic," "markedly problematic," "slightly problematic," and "no problem" were scored as 3, 2, 1, and 0 points, respectively. The total score ranges from 0 to 24 points. The cutoff value for discriminating sleep problems based on the ICD‐10 was 6 points (Soldatos et al., 2003). The reliability and validity of the AIS was verified; Cronbach's *α* coefficient for adolescents has been reported as .81 (Chung et al., 2011). In the present study, the *α* value was .82.

### 2.3 Ethics approval and consent to participate

This study was approved by the Ethics Review Committee of the Niigata University of Health and Welfare (17657). Consent was obtained from the individuals in charge of each participating school before conducting the survey. While recruiting participants, we clearly explained the research objective and method to the participants in writing, and that there would be respect for voluntary participation, freedom of consent and withdrawal, protection of personal information, and no disadvantages before they provided consent. Consent was obtained from both the parents and participants.

### 2.4 Data analysis

Means (standard deviations) are reported for continuous variables. Frequencies are reported for categorical variables. To evaluate any differences in the continuous variables between participants with and without ED, we confirmed the data distribution using the Kolmogorov–Smirnov test, and we used the Mann–Whitney *U* test because it was nonparametric. For categorical variables (sex and all AIS items), between‐groups differences were analyzed using *χ* ^2^ tests (with Yates’ correction) or Wilcoxon's rank‐sum test of order statistics. The effect size *r* was calculated by the formula *Z*/√*N* for the between‐group comparison. Spearman's correlation coefficient was used to check for correlations between each score of the EAT‐26 and AIS. To address type 1 errors in the analysis, the Benjamini–Hochberg (BH) method was used, with a false discovery rate of 0.05. The two‐tailed significance level was set at 5%. Statistical analyses were conducted using SPSS Statistics version 26 (IBM, Armonk, NY, USA).

## 3 RESULTS

### 3.1 Clinical characteristics of the participants

Participant characteristics are reported in Table 1. Questionnaires were distributed to 530 participants, of which 498 (94%) were collected. Of these, 100 were excluded from analysis due to missing information or the participant's age being >18 years, resulting in a total of 398 effective responses (75.1%).

**TABLE 1:** Clinical characteristics of noneating disorders (ED) and potential ED participants

	Non‐ED (*n* = 381)	Potential ED (*n* = 17)	*p* (BH)	Effect size
Age (years)	14.9 ± 1.7 (12–18)	16.0 ± 1.9 (12–18)	NS	0.12
Sex				
Male	199	6	NS	0.07
Female	182	11		
Height (cm)	161.4 ± 8.6 (136–187)	162.5 ± 8.3 (151–177)	NS	0.02
Weight (kg)	51.8 ± 10.2 (30–97)	52.7 ± 9.0 (39–72)	NS	0.03
BMI (kg/m^2^)	19.8 ± 3.2 (12.3–37.4)	19.9 ± 2.2 (15.8–24.0)	NS	0.04
BMI percentile	19.2 ± 1.3 (17.4–20.0)	19.7 ± 2.4 (17.2–24.0)	NS	0.09
AIS				
Total score	3.7 ± 3.2 (0–20)	7.1 ± 6.8 (0–20)	NS	0.08
Nocturnal score	2.3 ± 2.2 (0–12)	3.9 ± 4.1 (0–12)	NS	0.05
Daytime score	1.4 ± 1.4 (0–9)	3.2 ± 2.9 (0–9)	.042	0.12

*Note*: Values are expressed as mean ± SD (range). The Mann–Whitney *U* test indicates the effect size *r*, and the *χ*2 test indicates the effect size *φ*. BMI percentile is based on the 50th percentile by sex and age.

Abbreviations: AIS, Athens Insomnia Scale; BH, Benjamini–Hochberg method; BMI, body mass index; NS, not significant.

Of the 398 participants, 17 (4.3%) had an EAT‐26 total score of 20 or higher. Among boys, the rate was 6 of 199 participants (3%), and 11 of 182 among girls (6%). There were 89 participants (22.4%) with AIS scores ≥6, of which 36 (17.6%) were boys and 53 (27.5%) were girls.

No significant between‐group differences were found regarding age, sex, height, weight, BMI, or BMI percentile. Next, we investigated the EAT‐26 and AIS scores, which are the main outcome indicators of the study. Participants with potential ED showed significantly higher daytime functional vulnerability potentially associated with sleep problems than participants without such characteristics (*U* = 2158.5, *p* = .042), and a small effect size was found (*r* = 0.12).

### 3.2 Associations between potential ED and AIS items

We investigated the relationship between each item of the AIS and potential ED to further clarify the details of sleep problems (Table 2). No significant associations were found for adolescents with and without potential ED for sleep initiation, awakening, early morning awakening, total sleep duration, or overall quality. However, adolescents with potential ED had significantly more severe problems with items evaluating daytime functional vulnerability potentially associated with sleep problems (i.e., lower sense of well‐being and physical and mental functioning during the day) than adolescents without potential ED (*z* = 3.984, *p* < .001; and *z* = 2.836, *p* = .018). Furthermore, although the item concerning sleepiness during the day lost statistical significance after BH correction, it was significantly more severe in adolescents with potential ED before correction (*z* = 2.179, *p* = .029; and *p* = .078 after BH correction). Effect sizes were small for sense of well‐being, daytime physical and mental functioning, and daytime sleepiness (*r* = 0.20, *r* = 0.14, *r* = 0.11, respectively). These results suggest that adolescents with potential ED are not different from adolescents without ED with respect to sleep problems at night, but they do have psychological vulnerabilities during the day that are often attributed to sleep problems.

**TABLE 2:** Association of Athens Insomnia Scale (AIS) subdomains with high and low scorers on Eating Attitudes Test‐26 (EAT‐26)

		Non‐ED		Potential ED					
		*N*	%	*N*	%	*z*	*p* (unadjusted)	*p* (BH)	*r* (effect size)
Sleep initiation	No problem	201	52.8	6	35.3	1.559	.119	.191	0.08
	Slightly delayed	125	32.8	7	41.2				
	Markedly delayed	44	11.5	2	11.8				
	Very delayed or did not sleep at all	11	2.9	2	11.8				
Awakening during the night	No problem	324	85.0	13	76.5	0.968	.333	.381	0.05
	Minor problem	45	11.8	3	17.6				
	Considerable problem	11	2.9	1	5.9				
	Serious problem or did not sleep at all	1	0.3	0	0				
Early morning awakening	Not earlier	314	82.4	12	70.6	1.504	.133	.177	0.08
	A little earlier	58	15.2	2	11.8				
	Markedly earlier	7	1.8	1	5.9				
	Much earlier or did not sleep at all	2	0.5	2	11.8				
Total sleep duration	Sufficient	172	44.6	9	52.9	0.342	.733	.733	0.02
	Slightly insufficient	165	43.0	3	17.6				
	Markedly insufficient	38	10.4	2	11.8				
	Very insufficient or did not sleep at all	6	2.1	3	17.6				
Overall quality of sleep	Satisfactory	190	45.1	7	41.2	1.706	.088	.176	0.09
	Slightly unsatisfactory	160	42.0	4	23.5				
	Markedly unsatisfactory	25	6.6	4	23.5				
	Very unsatisfactory or did not sleep at all	6	1.6	2	11.8				
Sense of well‐being during the day	Normal	313	81.2	8	47.1	3.984	<.001***	<.001***	0.20
	Slightly decreased	61	16.0	4	23.5				
	Markedly decreased	2	0.5	3	17.6				
	Very decreased	5	1.3	2	11.8				
Functioning (physical and mental) during the day	Normal	315	82.7	10	58.8	2.836	.005**	.018*	0.14
	Slightly decreased	47	12.3	2	11.8				
	Markedly decreased	14	3.7	2	11.8				
	Very decreased	5	1.3	3	17.6				
Sleepiness during the day	None	103	27.0	4	23.5	2.179	.029*	.078	0.11
	Mild	207	54.3	4	23.5				
	Considerable	59	15.5	7	41.2				
	Intense	12	3.2	2	11.8				

Abbreviation: BH, Benjamini–Hochberg method.

* *p* < .05.

** *p* < .01.

*** *p* < .001.

### 3.3 Associations between ED symptoms and sleep problems

We analyzed the correlations between the EAT‐26 total, Dieting, Bulimia, and Oral Control scores and the AIS nocturnal and daytime scores to investigate the relationship between potential ED and sleep problems (Table 3). There were significant correlations between EAT‐26 scores and the AIS Nocturnal or Daytime scores as follows: Total score (nocturnal *r* = 0.265, 95% confidence interval [CI] = 0.169–0.357, *p* < .001; daytime *r* = 0.285, 95% CI = 0.189–0.376, *p* < .001), dieting score (nocturnal *r* = 0.253, 95% CI = 0.156–0.345, *p* < .001; daytime *r* = 0.254, 95% CI = 0.157–0.346, *p* < .001), bulimia score (nocturnal *r* = 0.160, 95% CI = 0.059–0.257, *p* = .002; daytime *r* = 0.232, 95% Cl = 0.134–0.325, *p* < .001), and oral control score (nocturnal *r* = 0.138, 95% CI = 0.038–0.236, *p* = .006; daytime *r* = 0.142, 95% CI = 0.041–0.240, *p* = .005). Although all the correlations were weak, they suggest that Japanese middle‐ and high‐school students with more severe ED characteristics are more susceptible to sleep problems.

**TABLE 3:** Correlation between Eating Attitudes Test‐26 (EAT‐26) and Athens Insomnia Scale (AIS) scores

	EAT‐26 total		Dieting score		Bulimia score		Oral control score	
	*r*	*p* (BH)	*r*	*p* (BH)	*R*	*p* (BH)	*r*	*p* (BH)
Nocturnal	0.265	<.001	0.253	<.001	0.160	.002	0.138	.006
Daytime	0.285	<.001	0.254	<.001	0.232	<.001	0.142	.005

Abbreviation: BH, Benjamini–Hochberg method.

## 4 DISCUSSION

The study found that about 4% of the sample had a potential ED. Further, adolescents with potential ED showed significantly increased vulnerability to functioning and sense of well‐being during the day on the AIS compared to adolescents without such characteristics. Significant positive correlations were also confirmed between the EAT‐26 subscales, which represent potential ED and sleep problems (AIS nocturnal and daytime scores). Our findings suggest that Japanese adolescents with potential ED are vulnerable to a reduced sense of well‐being and physical and mental functioning during the day, which are important indicators of sleep problems. Further, adolescents with more severe ED characteristics are more likely to have a higher degree of daytime psychological vulnerability potentially attributable to sleep problems.

### 4.1 Sleep problems in adolescents with potential ED

The study found that 3% of boys and 6% of girls had potential ED, with total scores of 20 points or higher on the EAT‐26, while 17.6% of boys and 27.5% of girls were judged to have sleep problems with scores of 6 points or higher on the AIS. These results are similar to those of previous studies (Bos et al., 2013; Nishizawa et al., 2003; Spruyt et al., 2005) and confirm that problems with eating and sleeping that are unique to adolescence are present among Japanese adolescents as well. This may be related to the fact that adolescents are inherently at risk of developing ED and are vulnerable to sleep problems, and both these are naturally triggered as they grow (Gradisar et al., 2011; Volpe et al., 2016). Longitudinal studies of ED in particular have shown a developmental trajectory from minor body image distortions and dieting behaviors that begin in early‐ to mid‐adolescence to more extreme weight loss behaviors and clinical disorders developing in mid‐ to late adolescence and early adulthood (Shisslak et al., 1995).

Moreover, adolescents with potential ED were found to be vulnerable to reduced daytime functioning potentially related to sleep problems (Table 1). In particular, the finding that sleep‐related problems experienced during the day—lower sense of well‐being, reduced physical and mental functioning, and sleepiness—were associated with potential ED is very interesting (Table 2) because the International Statistical Classification of Diseases and Related Health‐10 uses marked distress caused by sleep problems and/or interference with ordinary activities of daily living to assess insomnia; a lower sense of well‐being, reduced physical and mental functioning, and sleepiness are important indicators of distress. Chardon et al. (2016) suggested a mechanism by which daytime sleepiness increases the risk of disordered eating attitudes and behaviors in adolescents through internalizing symptoms such as anxiety and depression, regardless of age. In sleep disorders, a similar mechanism has been identified only in children under 12 years (Chardon et al., 2016). Therefore, it is possible that the relationship between ED symptoms and daytime sleep‐related problems is not a simple one but is influenced by several factors. The between‐group comparisons in this study did not find an association between sleep problems at night and potential ED, but specifically identified daytime functional problems related to sleep (Tables 1 and 2). We considered age as a factor influencing this result. Therefore, we conducted a partial correlation analysis including age as a control variable, but the statistical significance of the EAT‐26 and AIS scores did not change (Table S1). This supports the fact that daytime sleepiness is independent of age and related to disordered eating attitudes (Chardon et al., 2016). An additional explanation might be the fact that the participants were Japanese. Indeed, Asian Americans may report higher levels of certain ED symptoms than Western and other racial groups (Uri et al., 2021). In contrast, some reports suggest that ED symptoms are comparable regardless of race (Javaras et al., 2008; Monterubio et al., 2020). There is no consensus on racial differences in ED characteristics; therefore, racial/ethnic differences must be considered as a confounding variable in the future.

In the correlation between the EAT‐26 and AIS subscales, both subscales’ scores increased significantly with increasing EAT‐26 subdomain scores (Table 3). In other words, the higher the score on the potential ED subscales, the more severe the daytime functional vulnerabilities; this aligns with prior research. Specifically, a study of adolescents aged 8–17 years reported that sleep disorders and daytime sleepiness were positively correlated with ED attitudes (Chardon et al., 2016). Furthermore, a study of adult females aged 20–47 years found that sleep problems (insufficient sleep, sleeping poorly, problems falling asleep, feeling sleepy during work or free time, and disturbed sleep) were significantly associated with binge eating behaviors (Trace et al., 2012). It is not possible, however, to reveal in this study whether sleep problems lead to ED or vice versa, or whether it is interactional. In any case, the nature of the association between potential ED and sleep problems is complex and may involve diverse psychological, social, and biological mechanisms.

It is important to note that the results of between‐group comparisons in this study only confirmed the association between potential ED and the AIS subscales of daytime physical and mental functioning, sense of well‐being, and sleepiness during the day; adolescents with potential ED did not exhibit (statistically significantly) more nighttime sleep problems than adolescents without ED (Table 2). For example, the vulnerability to a lower sense of well‐being during the day may be more a function of ED characteristics than sleep problems. This is because people with ED are reported to have lower psychological well‐being than people without, and this correlates negatively with EAT scores (Tomba et al., 2014). Additionally, ED is well known to be comorbid with other psychiatric disorders (especially anxiety and mood disorders) (Ulfvebrand et al., 2015), and depression is strongly associated with well‐being (Wood & Joseph, 2010). In other words, poor daytime physical and mental functioning and daytime well‐being may be influenced by confounding variables that were unexamined in this study. Meanwhile, even though potential ED and nocturnal sleep problems did not correlate in the between‐group analysis (Tables 1 and 2), they were associated in the correlation analysis (Table 3). Moreover, the strength of the correlation between EAT‐26 and nocturnal and daytime sleep problems was comparable. We cannot rule out the possibility that the reason for the seemingly contradictory results of the between‐group and correlation analyses is, again, the effects of confounding variables and sample size bias as described above. The between‐group analysis in this study had a small sample size of adolescents who screened positive on the EAT‐26, and the effect sizes were small (Tables 1 and 2). Future research is needed to further examine the relationship between potential ED and sleep problems by equalizing the sample size between groups and conducting a test battery using multiple scales. To focus on daytime sleep‐related problems in particular, the Epworth Sleepiness Scale (Takegami et al., 2009), which is used worldwide to assess daytime sleepiness and has been translated into Japanese, may be helpful. In addition, the Psychological Well‐being Scale (Ryff, 1989), which assesses multiple dimensions of daytime well‐being, such as positive evaluation of self and sense of self‐determination, may reveal more sleep problems in people with potential ED.

### 4.2 Practical implications

The cutoff value for the EAT‐26 was 20 points in this study, but research has reported that a score of 11 points or higher indicates the possibility of binge eating (Ebrahim et al., 2019). Therefore, although this study conservatively states that one in 23 adolescents have potential ED, a larger proportion may be at risk. Indeed, a systematic review reported that the point prevalence of ED in the broadest sense was one in five women (19.4%) and one in seven men (13.8%) (Galmiche et al., 2019). However, it is difficult to compare the prevalence because multiple biases, such as differences in evaluation tools, classification, and evolution, affect the results. In any case, the need for support systems to address ED is apparent from these numbers alone. In particular, professionals involved in adolescent mental health issues should provide support to adolescents who have potential ED.

While some ED patients may be distressed by their symptoms and seek professional help, others may conceal their illness and avoid seeking treatment (Nakai et al., 2014). This tendency may be even more pronounced in those with potential ED, particularly adolescents. Further, our results suggest that adolescents with more severe ED characteristics were more likely to exhibit greater problems regarding daytime sense of well‐being, physical and mental functioning, and daytime sleepiness (Tables 1, 2, 3). Our results suggest that focusing on the severity of difficulty with daytime functioning related to sleep problems may indicate the need for support at an early stage and the degree of necessity for adolescents with potential ED, which is difficult to identify clinically. Considering that interventions for sleep disorders and daytime sleepiness may lead to improvements in potential ED in adolescents (Chardon et al., 2016), an approach that comprehensively integrates both sleep problems and potential ED may be an option for considering support for potential ED in adolescents.

### 4.3 Limitations

This study has some limitations. First, because the EAT‐26 and AIS are self‐reported scales, the results may be biased due to participants’ subjectivity. For example, regarding weight, it may be difficult to understand the appropriate weight for actual age in adolescents, who have rapid physical and mental developmental trajectories. To examine this issue, we calculated the average 50th BMI percentile of participants by sex and age (Table 1) and confirmed that it was comparable to the corresponding average BMI values of Japanese children (7–18 years, *n *= 4587) (Yin et al., 2020). Therefore, it is unlikely that there is a bias in the subjective assessment of BMI in this study, but it would be preferable to obtain data by actual measurement in the future. In addition, objective and quantitative research using measures such as polysomnography or electroencephalography to evaluate sleep is also needed. Second, this study only considered Japanese participants aged 12–18 years, which limits the generalizability of the results. Third, because this was a cross‐sectional study, causal relationships could not be determined. Longitudinal research is necessary to understand participants’ experiences more deeply. Fourth, the sample size of participants who screened positive on the EAT‐26 was small (*n *= 17); we cannot ignore the possibility that the analysis of between‐group differences may be biased. In addition, the study showed a rather low power of 0.71, considering the sample size (*n *= 398). Future studies with larger sample size and equal group sizes will be necessary. Fifth, the results obtained in this study do not cover confounding variables that may affect sleep problems, such as the presence of other psychiatric disorders and race. Sixth, missing information on participant characteristics was not included in the analysis. However, when data with complete participant characteristics were compared with data without complete participant characteristics, there was no difference in the presence or absence of statistical significance after BH correction (data not shown). There were items that showed significance in potential ED and nocturnal sleep problems, although it should be noted that these were before BH correction. Thus, it is possible that the missing information in this study may have selection bias or bias due to the physical and mental state of the participants, and further investigation may be necessary. Nonetheless, our findings revealed a piece of the puzzle that could help detect Japanese adolescents with potential ED symptoms.

## 5 CONCLUSION

Japanese adolescents with potential ED showed a significant association with reduced daytime functioning and sense of well‐being on AIS, which are important indicators of sleep problems, in the between‐group comparisons of this study. Conversely, significant associations with sleep disorders that focused on the night were not found. Correlation analysis showed that all subitems of the Dieting, Bulimia, and Oral Control scores, which represent potential ED, were found to have significant positive associations with both nighttime and daytime sleep problems in the AIS. These findings were considered similar to those of previous studies conducted in Western countries. In summary, the study suggested that professionals working toward adolescent mental health issues need an approach that comprehensively integrates both sleep problems and potential ED.

## CONFLICT OF INTEREST

The authors declare no conflict of interest.

## AUTHOR CONTRIBUTIONS

Takaharu Hirai designed the study, analyzed the data, and drafted the manuscript. Yuta Mitobe contributed to the data curation and writing. Hiromi Hirai provided statistical advice and contributed to data interpretation. Momoka Takeda and Mikiko Hayashi contributed to data interpretation. All authors have approved the manuscript.

### PEER REVIEW

The peer review history for this article is available at https://publons.com/publon/10.1002/brb3.2605.

## Supporting information

Supporting Information

## Data Availability

The datasets used and/or analyzed during the current study are available from the corresponding author upon reasonable request.

## References

[brb32605-bib-0001] Abebe, D. S. , Lien, L. , & von Soest, T. (2012). The development of bulimic symptoms from adolescence to young adulthood in females and males: A population‐based longitudinal cohort study. International Journal of Eating Disorders, 45(6), 737–745. 10.1002/eat.20950 22886952

[brb32605-bib-0002] Allison, K. C. , Spaeth, A. , & Hopkins, C. M. (2016). Sleep and eating disorders. Current Psychiatry Reports, 18(10), 92. 10.1007/s11920-016-0728-8 27553980

[brb32605-bib-0003] American Psychiatric Association . (2013). Diagnostic and statistical manual of mental disorders (5th ed.). American Psychiatric Association.

[brb32605-bib-0004] Asaad, T. A. , Esawy, H. I. , Abdel Razek Mohamed, G. , Hussein Ahmed, H. , Elhabiby, M. M. , Khalil, S. A. , & El‐Hawary, Y. A. (2018). Sleep profile in anorexia and bulimia nervosa female patients. Sleep Medicine, 48, 113–116. 10.1016/j.sleep.2018.03.032 29890462

[brb32605-bib-0005] Blank, M. , Zhang, J. , Lamers, F. , Taylor, A. D. , Hickie, I. B. , & Merikangas, K. R. (2015). Health correlates of insomnia symptoms and comorbid mental disorders in a nationally representative sample of US adolescents. Sleep, 38(2), 197–204. 10.5665/sleep.4396 25325502 PMC4288600

[brb32605-bib-0006] Bos, S. C. , Soares, M. J. , Marques, M. , Maia, B. , Pereira, A. T. , Nogueira, V. , Valente, J. , & Macedo, A. (2013). Disordered eating behaviors and sleep disturbances. Eating Behaviors, 14(2), 192–198. 10.1016/j.eatbeh.2013.01.012 23557819

[brb32605-bib-0007] Bulik, C. M. , Hebebrand, J. , Keski‐Rahkonen, A. , Klump, K. L. , Reichborn‐Kjennerud, T. , Mazzeo, S. E. , & Wade, T. D. (2007). Genetic epidemiology, endophenotypes, and eating disorder classification. International Journal of Eating Disorders, 40, S52–S60. 10.1002/eat.20398 17573683

[brb32605-bib-0008] Bulik, C. M. , Kleiman, S. C. , & Yilmaz, Z. (2016). Genetic epidemiology of eating disorders. Current Opinion in Psychiatry, 29(6), 383–388. 10.1097/YCO.0000000000000275 27532941 PMC5356465

[brb32605-bib-0009] Carskadon, M. A. (2011). Sleep in adolescents: The perfect storm. Pediatric Clinics of North America, 58(3), 637–647. 10.1016/j.pcl.2011.03.003 21600346 PMC3130594

[brb32605-bib-0010] Chardon, M. L. , Janicke, D. M. , Carmody, J. K. , & Dumont‐Driscoll, M. C. (2016). Youth internalizing symptoms, sleep‐related problems, and disordered eating attitudes and behaviors: A moderated mediation analysis. Eating Behaviors, 21, 99–103. 10.1016/j.eatbeh.2016.01.007 26826649

[brb32605-bib-0011] Chung, K. F. , Kan, K. K. , & Yeung, W. F. (2011). Assessing insomnia in adolescents: Comparison of Insomnia Severity Index, Athens Insomnia Scale and Sleep Quality Index. Sleep Medicine, 12(5), 463–470. 10.1016/j.sleep.2010.09.019 21493134

[brb32605-bib-0012] Ebrahim, M. , Alkazemi, D. , Zafar, T. A. , & Kubow, S. (2019). Disordered eating attitudes correlate with body dissatisfaction among Kuwaiti male college students. Journal of Eating Disorders, 7(1), 37. 10.1186/s40337-019-0265-z 31649822 PMC6805684

[brb32605-bib-0013] Fairweather‐Schmidt, A. K. , & Wade, T. D. (2015). Changes in genetic and environmental influences on disordered eating between early and late adolescence: A longitudinal twin study. Psychological Medicine, 45(15), 3249–3258. 10.1017/S0033291715001257 26134758

[brb32605-bib-0014] Faul, F. , Erdfelder, E. , Lang, A. G. , & Buchner, A. (2007). G*Power 3: A flexible statistical power analysis program for the social, behavioral, and biomedical sciences. Behavior Research Methods, 39(2), 175–191. 10.3758/bf03193146 17695343

[brb32605-bib-0015] Galmiche, M. , Déchelotte, P. , Lambert, G. , & Tavolacci, M. P. (2019). Prevalence of eating disorders over the 2000‐2018 period: A systematic literature review. American Journal of Clinical Nutrition, 109(5), 1402–1413. 10.1093/ajcn/nqy342 31051507

[brb32605-bib-0016] Garner, D. M. , & Garfinkel, P. E. (1979). The Eating Attitudes Test: An index of the symptoms of anorexia nervosa. Psychological Medicine, 9(2), 273–279. 10.1017/s0033291700030762 472072

[brb32605-bib-0017] Garner, D. M. , Olmstead, M. P. , & Polivy, J. (1983). Development and validation of a multidimensional eating disorder inventory for anorexia nervosa and bulimia. International Journal of Eating Disorders, 2(2), 15–34. 10.1002/1098-108X(198321)2:2<15::AID-EAT2260020203>3.0.CO;2-6

[brb32605-bib-0018] Gradisar, M. , Gardner, G. , & Dohnt, H. (2011). Recent worldwide sleep patterns and problems during adolescence: A review and meta‐analysis of age, region, and sleep. Sleep Medicine, 12(2), 110–118. 10.1016/j.sleep.2010.11.008 21257344

[brb32605-bib-0019] Holt, K. , & Ricciardelli, L. A. (2002). Social comparisons and negative affect as indicators of problem eating and muscle preoccupation among children. Journal of Applied Developmental Psychology, 23(3), 285–304. 10.1016/S0193-3973(02)00108-9

[brb32605-bib-0020] Izydorczyk, B. , Truong Thi Khanh, H. , Lizińczyk, S. , Sitnik‐Warchulska, K. , Lipowska, M. , & Gulbicka, A. (2020). Body dissatisfaction, restrictive, and bulimic behaviours among young women: A Polish‐Japanese comparison. Nutrients, 12(3), 666. 10.3390/nu12030666 PMC7146317 32121384

[brb32605-bib-0021] Javaras, K. N. , Laird, N. M. , Reichborn‐Kjennerud, T. , Bulik, C. M. , Pope, H. G. Jr. , & Hudson, J. I. (2008). Familiality and heritability of binge eating disorder: Results of a case‐control family study and a twin study. International Journal of Eating Disorders, 41(2), 174–179. 10.1002/eat.20484 18095307

[brb32605-bib-0022] Littleton, H. L. , & Ollendick, T. (2003). Negative body image and disordered eating behavior in children and adolescents: What places youth at risk and how can these problems be prevented? Clinical Child and Family Psychology Review, 6(1), 51–66. 10.1023/a:1022266017046 12659451

[brb32605-bib-0023] Maezono, J. , Hamada, S. , Sillanmäki, L. , Kaneko, H. , Ogura, M. , Lempinen, L. , & Sourander, A. (2019). Cross‐cultural, population‐based study on adolescent body image and eating distress in Japan and Finland. Scandinavian Journal of Psychology, 60(1), 67–76. https://org/10.1111/sjop.12485 30395688 10.1111/sjop.12485 PMC7379298

[brb32605-bib-0024] McCabe, M. P. , & Ricciardelli, L. A. (2003). Sociocultural influences on body image and body changes among adolescent boys and girls. The Journal of Social Psychology, 143(1), 5–26. 10.1080/00224540309598428 12617344

[brb32605-bib-0025] McFarlane, T. , McCabe, R. E. , Jarry, J. , Olmsted, M. P. , & Polivy, J. (2001). Weight‐related and shape‐related self‐evaluation in eating‐disordered and non‐eating‐disordered women. The International Journal of Eating Disorders, 29(3), 328–335. 10.1002/eat.1026 11262513

[brb32605-bib-0026] Mintz, L. B. , & O'Halloran, M. S. (2000). The Eating Attitudes Test: Validation with DSM‐IV eating disorder criteria. Journal of Personality Assessment, 74(3), 489–503. 10.1207/s15327752jpa7403_11 10900574

[brb32605-bib-0027] Monterubio, G. E. , Fitzsimmons‐Craft, E. E. , Balantekin, K. N. , Sadeh‐Sharvit, S. , Goel, N. J. , Laing, O. , Firebaugh, M. ‐L. , Flatt, R. E. , Cavazos‐Rehg, P. , Taylor, C. B. , & Wilfley, D. E. (2020). Eating disorder symptomatology, clinical impairment, and comorbid psychopathology in racially and ethnically diverse college women with eating disorders. International Journal of Eating Disorders, 53(11), 1868–1874. 10.1002/eat.23380 32918315 PMC7669650

[brb32605-bib-0028] Mukai, T. , Crago, M. , & Shisslak, C. M. (1994). Eating attitudes and weight preoccupation among female high school students in Japan. Journal of Child Psychology and Psychiatry, 35(4), 677–688. 10.1111/j.1469-7610.1994.tb01213.x 8040220

[brb32605-bib-0029] Nakai, Y. (2003). The validity of Eating Attitudes Test (EAT). Seishin Igaku, 45(2), 161–165.

[brb32605-bib-0030] Nakai, Y. , Nin, K. , & Noma, S. (2014). Eating disorder symptoms among Japanese female students in 1982, 1992 and 2002. Psychiatry Research, 219(1), 151–156. 10.1016/j.psychres.2014.05.018 24889844

[brb32605-bib-0031] Neumark‐Sztainer, D. , Wall, M. , Larson, N. I. , Eisenberg, M. E. , & Loth, K. (2011). Dieting and disordered eating behaviors from adolescence to young adulthood: Findings from a 10‐year longitudinal study. Journal of the American Dietetic Association, 111(7), 1004–1011. 10.1016/j.jada.2011.04.012 21703378 PMC3140795

[brb32605-bib-0032] Nishizawa, Y. , Kida, K. , Nishizawa, K. , Hashiba, S. , Saito, K. , & Mita, R. (2003). Perception of self‐physique and eating behavior of high school students in Japan. Psychiatry and Clinical Neurosciences, 57(2), 189–196. 10.1046/j.1440-1819.2003.01100.x 12667166

[brb32605-bib-0033] Okajima, I. , Nakajima, S. , Kobayashi, M. , & Inoue, Y. (2013). Development and validation of the Japanese version of the Athens Insomnia Scale. Psychiatry and Clinical Neurosciences, 67(6), 420–425. 10.1111/pcn.12073 23910517

[brb32605-bib-0034] Pike, K. M. , Hoek, H. W. , & Dunne, P. E. (2014). Cultural trends and eating disorders. Current Opinion in Psychiatry, 27(6), 436–442. 10.1097/yco.0000000000000100 25211499

[brb32605-bib-0035] Rosenvinge, J. H. , & Pettersen, G. (2015). Epidemiology of eating disorders, part I: Introduction to the series and a historical panorama. Advances in Eating Disorders, 3(1), 76–90. 10.1080/21662630.2014.898206

[brb32605-bib-0036] Ryff, C. D. (1989). Happiness is everything, or is it? Explorations on the meaning of psychological well‐being. Journal of Personality and Social Psychology, 57(6), 1069–1081. 10.1037/0022-3514.57.6.1069

[brb32605-bib-0037] Shisslak, C. M. , Crago, M. , & Estes, L. S. (1995). The spectrum of eating disturbances. International Journal of Eating Disorders, 18(3), 209–219. 10.1002/1098-108x(199511)18:3<209::aid-eat2260180303>3.0.co;2-e 8556017

[brb32605-bib-0038] Soldatos, C. R. , Dikeos, D. G. , & Paparrigopoulos, T. J. (2003). The diagnostic validity of the Athens Insomnia Scale. Journal of Psychosomatic Research, 55(3), 263–267. 10.1016/s0022-3999(02)00604-9 12932801

[brb32605-bib-0039] Spruyt, K. , O'Brien, L. M. , Cluydts, R. , Verleye, G. B. , & Ferri, R. (2005). Odds, prevalence and predictors of sleep problems in school‐age normal children. Journal of Sleep Research, 14(2), 163–176. 10.1111/j.1365-2869.2005.00458.x 15910514

[brb32605-bib-0040] Stice, E. , Marti, C. N. , Shaw, H. , & Jaconis, M. (2009). An 8‐year longitudinal study of the natural history of threshold, subthreshold, and partial eating disorders from a community sample of adolescents. Journal of Abnormal Psychology, 118(3), 587–597. 10.1037/a0016481 19685955 PMC2849679

[brb32605-bib-0041] Takegami, M. , Suzukamo, Y. , Wakita, T. , Noguchi, H. , Chin, K. , Kadotani, H. , Inoue, Y. , Oka, Y. , Nakamura, T. , Green, J. , Johns, M. W. , & Fukuhara, S. (2009). Development of a Japanese version of the Epworth Sleepiness Scale (JESS) based on item response theory. Sleep Medicine, 10(5), 556–565. 10.1016/j.sleep.2008.04.015 18824408

[brb32605-bib-0042] Tomba, E. , Offidani, E. , Tecuta, L. , Schumann, R. , & Ballardini, D. (2014). Psychological well‐being in out‐patients with eating disorders: A controlled study. International Journal of Eating Disorders, 47(3), 252–258. 10.1002/eat.22197 24123214

[brb32605-bib-0043] Trace, S. E. , Thornton, L. M. , Runfola, C. D. , Lichtenstein, P. , Pedersen, N. L. , & Bulik, C. M. (2012). Sleep problems are associated with binge eating in women. International Journal of Eating Disorders, 45(5), 695–703. 10.1002/eat.22003 22331832 PMC3357460

[brb32605-bib-0044] Ulfvebrand, S. , Birgegård, A. , Norring, C. , Högdahl, L. , & von Hausswolff‐Juhlin, Y. (2015). Psychiatric comorbidity in women and men with eating disorders results from a large clinical database. Psychiatry Research, 230(2), 294–299. 10.1016/j.psychres.2015.09.008 26416590

[brb32605-bib-0045] Uri, R. C. , Wu, Y. K. , Baker, J. H. , & Munn‐Chernoff, M. A. (2021). Eating disorder symptoms in Asian American college students. Eating Behaviors, 40, 101458. 10.1016/j.eatbeh.2020.101458 33307468 PMC7906921

[brb32605-bib-0046] Volpe, U. , Tortorella, A. , Manchia, M. , Monteleone, A. M. , Albert, U. , & Monteleone, P. (2016). Eating disorders: What age at onset?. Psychiatry Research, 238, 225–227. 10.1016/j.psychres.2016.02.048 27086237

[brb32605-bib-0047] Wood, A. M. , & Joseph, S. (2010). The absence of positive psychological (eudemonic) well‐being as a risk factor for depression: A ten year cohort study. Journal of Affective Disorders, 122(3), 213–217. 10.1016/j.jad.2009.06.032 19706357

[brb32605-bib-0048] Yin, X. , Yang, X. , Ji, L. , Song, G. , Wu, H. , Li, Y. , Sun, Y. , Bi, C. , Li, M. , Zhang, T. , Kato, H. , Akira, S. , & Haneda, S. (2020). Comparison of growth and nutritional status of Chinese and Japanese children and adolescents. Annals of Human Biology, 47(5), 425–433. 10.1080/03014460.2020.1766564 32892638

