# Supplementary material for: Potential eating disorder exhibited with daytime functional vulnerabilities associated with sleep problems in Japanese adolescents: A cross‐sectional study
Source: Brain Behav. 2022 May 9;12(6):e2605. doi: 10.1002/brb3.2605 (PMC9226844; doi:10.1002/brb3.2605)
Supplement: Supplementary file 1 — Supporting Information [file BRB3-12-e2605-s001.docx]

| Table S1 Correlation between EAT-26 and AIS scores adjusted for age as a control variable. | | | | | | | | | | | | |
| --- | --- | --- | --- | --- | --- | --- | --- | --- | --- | --- | --- | --- |
|  |  | EAT-26 total | |  | Dieting score | |  | Bulimia score | |  | Oral Control score | |
|  |  | *r*  (Partial correlation) | *p*  (BH) |  | *r*  (Partial correlation) | *p*  (BH) |  | *r*  (Partial correlation) | *p*  (BH) |  | *r*  (Partial correlation) | *p*  (BH) |
| Nocturnal |  | 0.291 | <.001 |  | 0.231 | <.001 |  | 0.294 | <.001 |  | 0.187 | 0.002 |
| Daytime |  | 0.297 | <.001 |  | 0.266 | <.001 |  | 0.342 | <.001 |  | 0.109 | 0.030 |
| *AIS: Athens Insomnia Scale, EAT-26: Eating Attitudes Test-26, BH: Benjamini-Hochberg method.* | | | | | | | | | | | | |
